# Decoding of exosome heterogeneity for cancer theranostics

**DOI:** 10.1002/ctm2.1288

**Published:** 2023-06-09

**Authors:** Rajib Dhar, Sukhamoy Gorai, Arikketh Devi, Raman Muthusamy, Athanasios Alexiou, Marios Papadakis

**Affiliations:** ^1^ Department of Genetic Engineering Cancer and Stem Cell Biology Laboratory SRM Institute of Science and Technology Kattankulathur India; ^2^ Department of Neurological Sciences Rush University Medical Center Chicago Illinois USA; ^3^ Department of Microbiology Centre for Infectious Diseases Saveetha Dental College Chennai India; ^4^ Department of Science and Engineering Novel Global Community Educational Foundation Hebersham Australia; ^5^ AFNP Med Wien Austria; ^6^ Department of Surgery II, University Hospital Witten‐Herdecke, Heusnerstrasse 40 University of Witten‐Herdecke Wuppertal Germany

**Keywords:** biomarkers, cancer, exosome, exosome heterogeneity, therapeutics

1

Extracellular Vesicles (EVs) discovery establishes a new milestone in cancer research. It explains cancer biology in a new way. EVs classified some significant subclasses such as macrovesicle (originated from plasma membrane), apoptotic bodies (originated from the plasma membrane and endoplasmic reticulum), and exosomes (originated from endosomes).^1^ The theranostic signature of exosomes in cancer healing is impressive, but its heterogeneity leads most complicated domain of research. Therefore we comment on decoding exosome heterogeneity for future efficiency and effective cancer theranostics development. EVs are the cell‐secreted one of the most important entities. The exosome attracted much attention due to its versatile uses. Overall, its participation in intercellular communication and its inner cargos (DNA, RNA, proteins, lipids and glycan)^2^ can reprogram the recipient cells. In addition, it also shows the healthy or pathologic complication status of the parent cells. In cancer, tumor and exosomes interlink a complex chapter of cancer biology. Exosomes play an important role in accelerated tumor growth, immune cell reprogramming,^2^ angiogenesis,^3^ extracellular matrix remodelling,^4^, ^5^ metastasis,^4^, ^5^ epithelial‐mesenchymal transition (EMT),^4^, ^5^ organ‐specific metastasis,^5^, ^6^ drug resistance and cancer stem cell development.^3^, ^4^ Clinically aspect, the exosome is the source of the cancer biomarkers.^7^, ^8^, ^9^ Exosome heterogeneity^1^ is evolving into a major conflict in cancer biomarker and therapeutic research.^10^ The exosome heterogeneity (Figure 1) is based on several facts such as origin, size, quantity, and internal molecular diversity.^1^ Exosome size distribution is the most complicated side of tumor‐derived exosomes (TEXs). Scientific evidence shows that the same cells release differently‐sized exosomes. This size‐oriented biodiversity is reflected in using different isolation methods.^9^, ^10^ The tumor cells release more exosomes compared to healthy cells. The higher release of exosomes from tumor cells depends on several factors, such as drug and therapeutic exposures, hypoxic conditions, and senescence. The functional diversity of exosomes is related to the surface molecular expression and inner cargos of exosomes. All these complicated/complex parameters lead to a challenging ecosystem development of exosome‐based cancer theragnostics (combination of biomarkers and therapeutics research). This scenario requires a solution to solve these challenges. Based on several innovative nano platform‐based approaches transform these challenges into an opportunity for exosome researcher in a detailed exploration of exosome.^11^ Exosomes isolation, size and quantity related complication solved via microfluidic device, fluorescence antibody based exosome tracking, Nanoparticle tracking analysis (NTA) (above 60 nm exosome size challenging for its application),^12^, ^13^ magnetic and surface plasma resonance principle based single exosome screening,^1^ advanced sensitive flow cytometry (it is capable of the separation of exosome subpopulation) and electrophoresis chip. Molecular profiling of exosomes is one of the most critical tasks revealing the functional diversity of bioactive cargo molecules of exosomes. Droplet digital polymerase chain reaction (it is sensitive to the detection of rear mutations in tumor‐derived exosomes),^1^ microfluidic technology combined with a chip system, and an electrochemical principle‐based micro RNA profiling of exosomes explain the cancer complication of a new dimension.^1^ The downstream process of exosome profiling needs a muti‐omics approach for proper molecular diversity to understand.^1^ The single cells exosome profiling approach combines with machine learning for more precision cancer marker development.^14^ All these technological advancements support constructing the next‐generation promising cancer theranostic era based on exosomes.^15^, ^16^ Finally, decoding exosome heterogeneity opens a new door for exosome‐based precision medicine^17^ and vaccines^18^ for cancer. We hope this article encourages large‐scale EV research to explore a single exosome profiling approach and future exosome‐based cancer precision medicine development.

**FIGURE 1:**
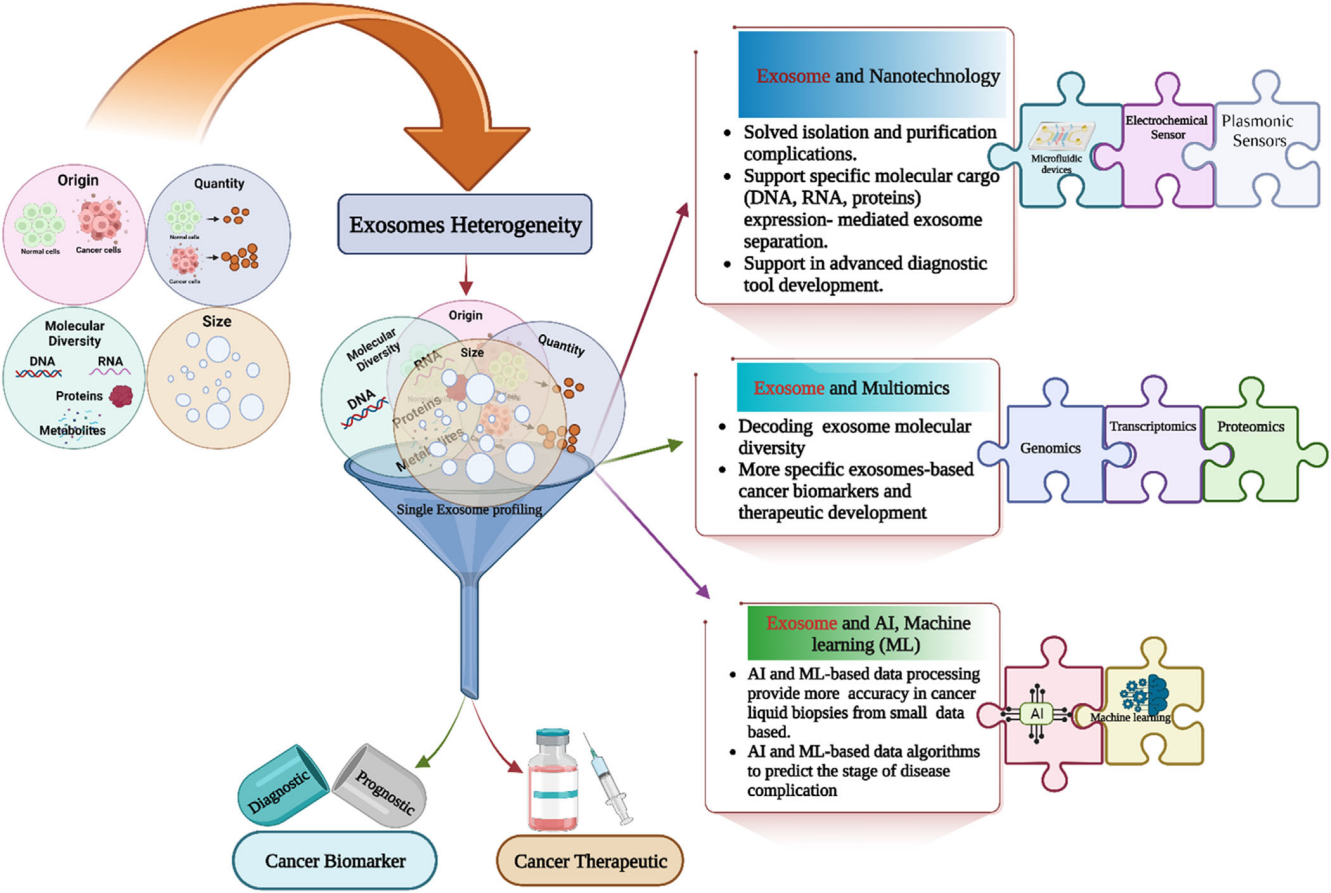
Exosome heterogeneity and single exosome profiling (created with Biorender.com).

## CONFLICT OF INTEREST STATEMENT

The authors declare no conflict of interest.

## FUNDING INFORMATION

There is no funding for this study.
